# Risk of non-Hodgkin's lymphoma following tuberculosis

**DOI:** 10.1054/bjoc.2000.1551

**Published:** 2001-01-01

**Authors:** J Askling, A Ekbom

**Affiliations:** Department of Medical Epidemiology, Karolinska Institute, Box 281, Stockholm, S-171 77, Sweden; Department of Epidemiology, Harvard School of Public Health, Boston, MA, USA

**Keywords:** non-Hodgkin's lymphoma, tuberculosis, cohort, Sweden

## Abstract

To study the association between chronic infections and non-Hodgkin's lymphoma (NHL), we assessed the risk of NHL in a Swedish cohort of 5050 individuals with tuberculosis 1939–1960. The overall relative risk was moderately increased, largely accounted for by high risks following severe tuberculosis diagnosed a long time ago. © 2001 Cancer Research Campaign http://www.bjcancer.com


*British Journal of Cancer* (2001) **84**(1), 113–115
© 2001 Cancer Research Campaign
doi: 10.1054/ bjoc.2000.1551, available online at http://www.idealibrary.com on IDEAL®

http://www.bjcancer.com

Short Communication

### Risk of non-Hodgkin's lymphoma following tuberculosis

**J Askling[1] and A Ekbom[1,2]**

[1]Department of Medical Epidemiology, Karolinska Institute, Box 281, S-171 77 Stockholm, Sweden; [2]Department of Epidemiology, Harvard School of Public Health, Boston, MA, USA

**Summary** To study the association between chronic infections and non-Hodgkin's lymphoma (NHL), we assessed the risk of NHL in a Swedish cohort of 5050 individuals with tuberculosis 1939–1960. The overall relative risk was moderately increased, largely accounted for by high risks following severe tuberculosis diagnosed a long time ago. © 2001 Cancer Research Campaign http://www.bjcancer.com

**Keywords**: non-Hodgkin's lymphoma; tuberculosis; cohort; Sweden

Over the past decades, the incidence of non-Hodgkin's lymphoma (NHL) has increased in many western countries (Devesa and Fears, 1992) for reasons that are largely unknown. Chronic inflammation has been linked to increased occurrence of NHL (Holmes et al, 1989; Baecklund et al, 1998). Several moderate-sized case-control studies of patients with NHL have found associations with histories of chronic infections including tuberculosis (Bernard et al, 1984; Franceschi et al, 1989; Doody et al, 1992; La Vecchia et al, 1992; Tavani et al, 2000). During the first half of the 20th century, the incidence and case-fatality of tuberculosis decreased markedly (The Swedish Institute for Infectious Disease Control, 1989), in the same birth cohorts that have later experienced an increasing incidence of NHL. Based on this ecological correlation, and on the positive association between the two diseases reported in case-control studies, we hypothesized that individuals with a history of tuberculosis would be at increased risk of NHL.

At two Swedish tuberculosis dispensaries and one sanatorium with population-based catchment areas, we identified 5199 individuals consecutively diagnosed or admitted with tuberculosis in the years 1939–1960. For each individual, we collected information on date of birth, sex, date of entry (diagnosis or admission), location of tuberculosis (pulmonary vs extrapulmonary), result of sputum microsopy on admission (sanatorium patients up to 1953 only), admission to thoracic surgery (sanatorium patients up to 1953 only), and socioeconomic status (white collar, blue collar, unskilled worker, or missing, categorized on the basis of the occupation of the head of the family). The cohort was linked to the population-based Swedish cancer register for 1958–1996 (Mattsson and Wallgren, 1984), cause of death register for 1952–1996, and to the population register. 139 individuals were lost to follow-up. Ten individuals were excluded due to data irregularities precluding follow-up, leaving 5050 individuals (2680 males and 2370 females) in the cohort. Of these, 3353 individuals entered 1939–1952 and 1697 entered 1953–1960. Standardized all-cause mortality ratios (SMR), and standardized incidence ratios (SIR) of malignancies at all sites, of NHL, and of Hodgkin's disease were calculated by dividing the observed number of events with that expected according to sex-, age- and calendar period-specific rates. Start of follow-up was set to last of: date of diagnosis of tuberculosis or 1 January 1952 (SMR) or 1958 (SIR). End of follow-up was set to date of death, emigration, or 31 December 1996, whichever occurred first. 95% confidence intervals (95% CI) were calculated assuming a Poisson-distribution for the observed cases (Breslow and Day, 1987). Poisson-regression was used to compare risk estimates (Breslow and Day, 1987).

2797 deaths were registered during 163 270 person-years of follow-up, the SMR being 1.40 (95% CI = 1.35–1.46). The SMR declined with time since tuberculosis, and was 1.17 (95% CI = 1.07–1.27) after 40 or more years since tuberculosis ($P$ for linear trend < 0.0001). Individuals who entered the cohort 1939–1952 had a significantly higher SMR than those who entered 1953–1960 (adjusted for time since tuberculosis, data not shown). Admission (vs no admission) to thoracic surgery, positive (vs negative) direct microscopy, extrapulmonary (vs pulmonary) tuberculosis, and low (vs high) socioeconomic status were each associated with significantly higher SMRs (data not shown). 1073 cancers were registered and the SIR was 1.20 (95% CI = 1.13–1.28). The all-site SIRs were similar in all latency periods and did not vary significantly with calendar period of entry, admission to thoracic surgery, sputum smear status, location of tuberculosis, or socioeconomic status (data not shown). A total of 33 cases of NHL were registered and the SIR was 1.42 (95% CI = 0.98–1.99) (Table 1). The relative risk was highest after 40 or more years following entry (SIR = 2.25, 95% CI = 1.23–3.78, $n$ = 14). The SIRs were close to unity in each 5-year interval up to 40 years since the tuberculosis, but above 2.0 in each subsequent interval (data not shown). Individuals who entered the cohort 1939–1952 had a significantly higher risk (SIR = 1.77, 95% CI = 1.16–2.57, $n$ = 27) than those who entered 1953–1960, whose risk was not elevated (Table 1). When adjusted for each other, the elevated risks associated with more than 40 years of latency, and with entry during 1939–1952, remained largely similar yet no longer significant. Male sex, positive (vs negative) sputum smear, and admission (vs no admission) to thoracic surgery were associated with non-significantly higher SIRs, and the risks were similar for pulmonary and extrapulmonary tuberculosis (data not shown). However, 14 of the 33 cases of NHL occurred among individuals with either positive sputum smear or admission to

*Received 23 June 2000*
*Revised 2 October 2000*
*Accepted 2 October 2000*

*Correspondence to:* J Askling. E-mail: johan.askling@mep.ki.se

**Table 1** Standardized incidence ratio (SIR) of non-Hodgkin's lymphoma 1958–1996 with 95% confidence interval (95% CI) and number of observed cases (*n*) by calendar period of entry, severity of tuberculosis (defined by sputum smear status and admission to thoracic surgery) and time since tuberculosis, in a cohort of 5050 Swedish patients with tuberculosis diagnosed 1939–1960 and followed up until 1997

| | | SIR (95% CI) *n* | | |
|---|---|---|---|---|
| **Period of entry** | **Subjects** | **0–39 years since TB** | **40+ years since TB** | **All latency intervals** |
| 1939–1952 | All | 1.38 (0.73–2.34) 13 | 2.42 (1.33–4.07) 14 | 1.77 (1.16–2.57) 27[a] |
| | Smear positive or operated | 1.48 (0.48–3.46) 5 | 5.34 (2.44–10.1) 9[b,c] | 2.77 (1.51–4.65) 14[d] |
| | No information | 2.22 (0.72–5.17) 5 | 3.70 (0.76–10.8) 3 | 2.61 (1.13–5.14) 8 |
| | Smear negative, not operated | 0.77 (0.16–2.26) 3 | 0.61 (0.07–2.20) 2 | 0.70 (1.23–1.63) 5 |
| 1953–1960 | All | 0.79 (0.29–1.73) 6 | 0.00 (0.00–8.52) 0 | 0.75 (0.27–1.63) 6 |
| Both periods | All | 1.11 (0.67–1.74) 19 | 2.25 (1.23–3.78) 14[e] | 1.42 (0.98–1.99) 33 |

[a]*P* for difference compared to entry in 1953–1960 = 0.04; [b]*P* for difference compared to 0–39 years since tuberculosis = 0.02; [c]*P* for linear trend from no, through missing, to yes = 0.001; [d]*P* for difference compared to individuals with negative sputum smear and not operated on = 0.004; [e]*P* for difference compared to 0–39 years since tuberculosis = 0.05

surgery (SIR = 2.77, 95% CI = 1.51–4.65), compared to five among individuals not admitted to surgery and with negative sputum microscopy (SIR = 0.70, 95% CI = 0.23–1.63), and 14 among individuals with no information on either characteristic (SIR = 1.26, 95% CI = 0.69–2.12, *P* for linear trend over estimates = 0.003). Among those smear-positive or admitted to surgery, the risk was significantly higher after 40 or more years since tuberculosis (SIR = 5.34, 95% CI = 2.44–10.1, *n* = 9) compared to 0–39 years since tuberculosis (Table 1). Alternative categorizations of latency time resulted in a similar risk pattern. The SIR of Hodgkin's disease was not significantly elevated (SIR = 1.65, 95% CI = 0.71–3.26, *n* = 8) but displayed a similar pattern as NHL with the highest point estimate 40 or more years since the tuberculosis (SIR = 4.18, 95% CI = 0.51–15.1, *n* = 2).

This study indicates that the risk of NHL following tuberculosis is increased, but only among individuals diagnosed with tuberculosis before 1953, and among them, in particular among individuals with severe tuberculosis. The increased overall risk of NHL corroborates the results of several case-control studies of patients with NHL (Bernard et al, 1984; Franceschi et al, 1989; Doody et al, 1992; La Vecchia et al, 1992; Tavani et al, 2000), although some have found no association (Tielsch et al, 1987; Cartwright et al, 1988). None of the case-control studies have, however, included characteristics of the tuberculosis. Two cohort-studies (performed for other purposes) of patients with tuberculosis analysed the risk for NHL (Howe et al, 1979; Davis et al, 1989) but neither found a positive association. However, in one study patients were followed for only 23 years, and proportionate mortality ratios were used which are inherently difficult to interpret in the presence of competing mortality (Howe et al, 1979). The other found an increased overall risk of dying from Hodgkin's disease but no increased risk for the combined group of non-Hodgkin's lymphoma, multiple myeloma and polycythaemia vera. No separate analysis of non-Hodgkin's lymphoma was performed (Davis et al, 1989). No analysis of latency was presented. An increased risk for NHL may thus well have been concealed in both these studies.

Patients with tuberculosis may differ from the general population with respect to several factors, such as socioeconomic status, use of tobacco, alcohol and coffee, exposure to ionizing radiation and isoniazid, and body-mass index. Since none of these factors have been consistently identified as risk factors for NHL (Stott et al, 1976; Franceschi et al, 1989; Devesa and Fears, 1992; La Vecchia et al, 1992; Ron et al, 1994; Tavani et al, 1994; Nelson et al, 1997; Adami et al, 1998; Holly et al, 1999; Zhang et al, 1999), substantive confounding by these factors is unlikely. Surveillance bias, that is, if individuals with a history of tuberculosis received closer medical surveillance than healthy individuals, would more likely have resulted in generally increased risks rather than the observed calendar period-dependent risks. The classification of sputum smear status, location of tuberculosis, and surgical treatment does not take into consideration later relapses. Bias resulting from such misclassification would, however, attenuate rather than create differences between exposure-groups, and is thus an unlikely explanation for the observed variations in risk. The lack of information on severity during the 1953–1960 period of entry precludes a more detailed risk-analysis within this interval. However, it does not change the absence of overall risk increase during the interval, although the low number of expected cases in the long latency interval during this period makes it difficult to completely exclude a long-term effect. Validation-studies have found that one third of the registered cases of Hodgkin's disease during the 1970s were misclassified NHL (Martinsson et al, 1992). We therefore also assessed the risk of Hodgkin's disease. The similarity of the SIRs for NHL and for Hodgkin's disease may be due to misdiagnosis of the former as the latter.

Severe tuberculosis (defined as individuals with positive sputum smear or admission to surgery, who had the highest overall mortality) was associated with a markedly increased risk of NHL. In contrast, individuals diagnosed after 1952, when curative chemotherapy (isoniazid) was introduced, were not at increased risk of NHL. Inflammatory intensity and duration are important risk determinants for malignant lymphomas complicating rheumatoid arthritis and coeliac disease (Holmes et al, 1989; Baecklund et al, 1998) and our results suggest that these factors also may influence the association between tuberculosis and NHL: limited infection did not increase NHL risk (tuberculosis diagnosed after 1952 or uncomplicated tuberculosis diagnosed 1939–1952), while the highest inflammatory load was associated with the highest risk increase (long-standing, severe tuberculosis diagnosed 1939–1952).

There is evidence of genetic variation in the susceptibility to tuberculosis, through genes encoding components of the inflammatory response (Bellamy et al, 1998). Since it has been proposed that low-secretor genotypes of TNF-alpha are associated with increased susceptibility to follicular NHL (Fitzgibbon et al, 1999), an alternative interpretation of our results is that NHL is a result of increased susceptibility rather than a consequence of tuberculosis itself.

 © 2001 Cancer Research Campaign

In conclusion, this study suggests that the risk of NHL is increased in individuals with a history of tuberculosis, but only among individuals with tuberculosis, particularly severe, diagnosed a long time ago. Whether the observed risk increase is due to the tuberculosis itself, an underlying susceptibility, or associated exposures is not clear. Our results raise the question whether one reason behind the increasing incidence of NHL over the past decades is the increasing survival into older age groups of individuals with what used to be lethal infections of early adulthood, such as tuberculosis.

### ACKNOWLEDGEMENTS

This study was supported by grants from the Swedish Heart Lung Foundation and from the National Heart and Lung Association.

### REFERENCES

Adami J, Nyren O, Bergstrom R, Ekbom A, Engholm G, Englund A and Glimelius B (1998) Smoking and the risk of leukemia, lymphoma, and multiple myeloma (Sweden). *Cancer Causes Control* **9**: 49–56

Baecklund E, Ekbom A, Sparen P, Feltelius N and Klareskog L (1998) Disease activity and risk of lymphoma in patients with rheumatoid arthritis: nested case-control study. *BMJ* **317**: 180–181

Bellamy R, Ruwende C, Corrah T, McAdam KP, Whittle HC and Hill AV (1998) Variations in the nrampl gene and susceptibility to tuberculosis in West Africans. *N Engl J Med* **338**: 640–644

Bernard SM, Cartwright RA, Bird CC, Richards ID, Lauder I and Roberts BE (1984) Aetiologic factors in lymphoid malignancies: a case-control epidemiological study. *Leuk Res* **8**: 681–689

Breslow NE and Day NE (1987) Statistical methods in cancer research. Volume II – The design and analysis of cohort studies. *IARC Sci Publ* 1–406

Cartwright RA, McKinney PA, O'Brien C, Richards ID, Roberts B, Lauder I, Darwin CM, Bernard SM and Bird CC (1988) Non-Hodgkin's lymphoma: case control epidemiological study in Yorkshire. *Leuk Res* **12**: 81–88

Davis FG, Boice Jr JD, Hrubec Z and Monson RR (1989) Cancer mortality in a radiation-exposed cohort of Massachusetts tuberculosis patients. *Cancer Res* **49**: 6130–6136

Devesa SS and Fears T (1992) Non-Hodgkin's lymphoma time trends: United States and international data. *Cancer Res* **52**: 5432s–5440s

Doody MM, Linet MS, Glass AG, Friedman GD, Pottern LM, Boice Jr JD and Fraumeni JF Jr (1992) Leukemia, lymphoma, and multiple myeloma following selected medical conditions. *Cancer Causes Control* **3**: 449–456

Fitzgibbon J, Grenzelias D, Matthews J, Lister TA and Gupta RK (1999) Tumour necrosis factor polymorphisms and susceptibility to follicular lymphoma. *Br J Haematol* **107**: 388–391

Franceschi S, Serraino D, Bidoli E, Talamini R, Tirelli U, Carbone A and La Vecchia C (1989) The epidemiology of non-Hodgkin's lymphoma in the north-east of Italy: a hospital-based case-control study. *Leuk Res* **13**: 465–472

Holly EA, Lele C, Bracci PM and McGrath MS (1999) Case-control study of non-Hodgkin's lymphoma among women and heterosexual men in the San Francisco Bay Area, California. *Am J Epidemiol* **150**: 375–389

Holmes GK, Prior P, Lane MR, Pope D and Allan RN (1989) Malignancy in coeliac disease – effect of a gluten-free diet. *Gut* **30**: 333–338

Howe GR, Lindsay J, Coppock E and Miller AB (1979) Isoniazid exposure in relation to cancer incidence and mortality in a cohort of tuberculosis patients. *Int J Epidemiol* **8**: 305–312

La Vecchia C, Negri E and Franceschi S (1992) Medical history and the risk of non-Hodgkin's lymphomas. *Cancer Epidemiol Biomarkers Prev* **1**: 533–536

Martinsson U, Glimelius B and Sundstrom C (1992) Lymphoma incidence in a Swedish county during 1969–1987. *Acta Oncol* **31**: 275–282

Mattsson B and Wallgren A (1984) Completeness of the Swedish Cancer Register. Non-notified cancer cases recorded on death certificates in 1978. *Acta Radiol Oncol* **23**: 305–313

Nelson RA, Levine AM, Marks G and Bernstein L (1997) Alcohol, tobacco and recreational drug use and the risk of non-Hodgkin's lymphoma. *Br J Cancer* **76**: 1532–1537

Ron E, Preston DL, Mabuchi K, Thompson DE and Soda M (1994) Cancer incidence in atomic bomb survivors. Part IV: Comparison of cancer incidence and mortality. *Radiat Res* **137**: S98–112

Stott H, Peto J, Stephens R and Fox W (1976) An assessment of the carcinogenicity of isoniazid in patients with pulmonary tuberculosis. *Tubercle* **57**: 1–15

Tavani A, Negri E, Franceschi S, Talamini R and La Vecchia C (1994) Coffee consumption and risk of non-Hodgkin's lymphoma. *Eur J Cancer Prev* **3**: 351–356

Tavani A, La Vecchia C, Franceschi S, Serraino D and Carbone A (2000) Medical history and risk of Hodgkin's and non-Hodgkin's lymphomas. *Eur J Cancer Prev* **9**: 59–64

The Swedish Institute for Infectious Disease Control (1989) *The Swedish Tuberculosis Index*. National Heart and Lung Foundation: Stockholm

Tielsch JM, Linet MS and Szklo M (1987) Acquired disorders affecting the immune system and non-Hodgkin's lymphoma. *Prev Med* **16**: 96–106

Zhang S, Hunter DJ, Rosner BA, Colditz GA, Fuchs CS, Speizer FE and Willett WC (1999) Dietary fat and protein in relation to risk of non-Hodgkin's lymphoma among women. *J Natl Cancer Inst* **91**: 1751–1758

*© 2001 Cancer Research Campaign*

